# Cancer metastasis through the prism of epithelial‐to‐mesenchymal transition in circulating tumor cells

**DOI:** 10.1002/1878-0261.12081

**Published:** 2017-06-09

**Authors:** Douglas S. Micalizzi, Daniel A. Haber, Shyamala Maheswaran

**Affiliations:** ^1^ Massachusetts General Hospital Cancer Center Harvard Medical School Charlestown MA USA; ^2^ Department of Medicine Harvard Medical School Charlestown MA USA; ^3^ Howard Hughes Medical Institute Chevy Chase MD USA; ^4^ Department of Surgery Harvard Medical School Charlestown MA USA

**Keywords:** cancer, circulating tumor cells, epithelial to mesenchymal transition, metastasis

## Abstract

Metastasis of epithelial cancer cells to distant sites is a particularly critical stage of cancer progression that typically marks the incurability of the disease. It is governed by a complex series of events including invasion and intravasation of tumor cells into the stroma and blood, respectively. Epithelial‐to‐mesenchymal transition (EMT), a phenotypic change marked by the loss of epithelial characteristics and the acquisition of invasive mesenchymal properties, is implicated in the dissemination of tumor cells. Circulating tumor cells (CTCs), the precursors of metastasis, can be used to interrogate the contribution of EMT in metastasis and therapeutic responses. The analysis of these CTCs and in particular the presence of inter‐ and intrapatient heterogeneity for markers of EMT has provided new insights into the metastatic process. This review will focus on epithelial–mesenchymal plasticity in CTCs and its potential clinical implications.

**Abbreviations**
CTCs: circulating tumor cells
EGFR: epidermal growth factor receptor
EMT: epithelial‐to‐mesenchymal transition
KRT: keratin
MET: mesenchymal‐to‐epithelial transition
RNA‐ISH: RNA *in situ* hybridization

## 1 Introduction

Epithelial‐to‐mesenchymal transition (EMT) is a tightly regulated lineage change occurring during gastrulation, neural crest delamination, and heart valve formation, developmental processes involved in embryonic patterning (Savagner, 2015). Hallmarks of EMT include the loss of epithelial markers and the acquisition of mesenchymal markers, disruption of cell–cell junctions, and apico‐basal polarity as well as remodeling of the cytoskeleton. These changes are associated with increased cell migration and resistance to anoikis (Savagner, 2015) and are triggered by several growth factors, cytokines, and numerous transcription factors. EMT is a reversible process that is dependent on the continued presence of the EMT‐inducing signal, the removal of which results in the reversion of the mesenchymal cell to an epithelial state [mesenchymal‐to‐epithelial transition (MET)].

In adult organisms, EMT is activated during physiologic and pathophysiologic responses including wound healing and fibrosis (Stone *et al*., 2016). EMT has been extensively implicated in cancer progression, specifically in promoting the early stages of metastasis, which involve invasion of tumor cells into the surrounding stroma and blood‐borne dissemination of cells to the lung, liver, bone, and brain (Nieto *et al*., 2016; Thiery *et al*., 2009). Once epithelial cancer cells, which have transitioned to a mesenchymal state, reach distant sites, it is postulated that they revert to the epithelial lineage through the process of MET (Gunasinghe *et al*., 2012). Tumor cells that are able to survive within the new microenvironment can acquire the ability to proliferate and establish metastatic tumors, which in most cancers marks a stage in which the cancer becomes incurable. In addition to a role in tumor cell invasion and dissemination, EMT of epithelial tumor cells is also linked to the acquisition of stem‐like characteristics and drug resistance (Aktas *et al*., 2009; Arumugam *et al*., 2009; Jolly *et al*., 2015; Marchini *et al*., 2013; Sequist *et al*., 2011; Witta *et al*., 2006). Interestingly, lineage tracing of epithelial and mesenchymal tumor cells within genetically engineered mice shows that EMT may not be a prerequisite for metastasis, but instead contributes to drug resistance, suggesting additional functions for EMT in cancer progression (Fischer *et al*., 2015; Maheswaran and Haber, 2015; Zheng *et al*., 2015).

**Figure 1:**
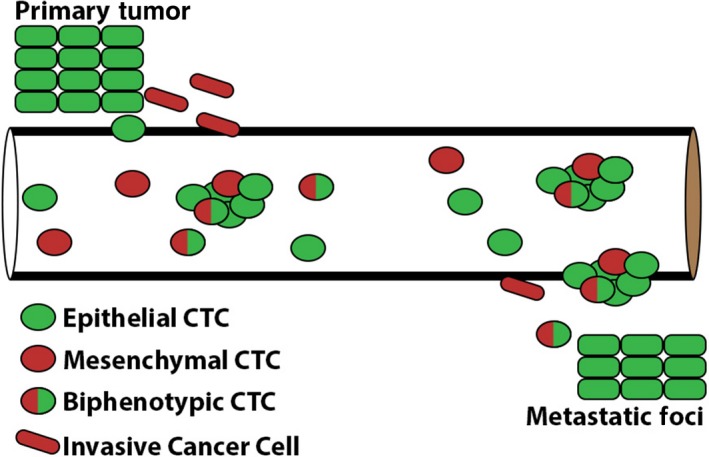
Epithelial‐to‐mesenchymal transition (EMT) in circulating tumor cells (CTCs): Invasive cells from the primary tumor exhibit epithelial plasticity including the loss of epithelial markers and gain of mesenchymal markers. Solitary and clusters of CTCs demonstrate heterogeneity of epithelial and mesenchymal markers.

Cancer cells co‐opting EMT to migrate and invade provides an attractive model to understand the critical steps involved in the initiation of metastasis, a highly complex process. However, identifying, within the primary tumor, epithelial tumor cells converting to a mesenchymal state has been complicated by the presence of stromal cells which express high levels of mesenchymal markers. As such, despite the dramatic invasive and tumorigenic phenotypes observed in mouse xenografts expressing master EMT‐inducing transcriptional regulators, Snail, Twist, and Slug among others (Ocana *et al*., 2012; Tran *et al*., 2014; Yang *et al*., 2004; Ye *et al*., 2015), the direct observation of EMT in the metastasis of human epithelial cancers has remained elusive. Increased invasion of tumor cells undergoing EMT suggests that mesenchymal characteristics would be prevalent in tumor cells entering the circulation. EMT of tumor cells in the blood has been evaluated by PCR analysis of peripheral blood mononuclear cells isolated from patients with cancer (Aktas *et al*., 2009; Lasa *et al*., 2013), but this approach has been unreliable due to the high rate of false signals detected in unpurified blood components. Analysis of circulating tumor cells (CTCs), precursors of metastasis which circulate in the blood as either individual tumor cells or as tumor cell clusters/tumor emboli, offers a rare opportunity to gain insight into the spread of cancer at a critical stage of metastasis (Maheswaran and Haber, 2010). Accumulating evidence from the analysis of these isolated CTCs has demonstrated significant heterogeneity of EMT markers supporting the concept of EMT as an important feature of invasive cancer cells (Fig. 1).

**Figure 2:**
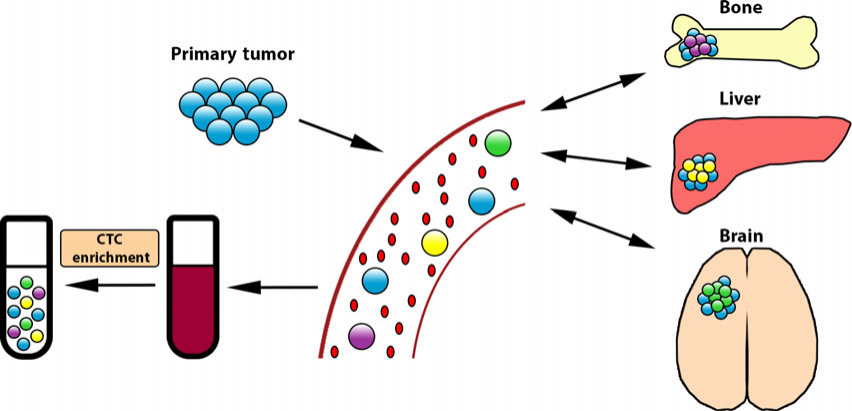
Heterogeneity of CTCs in metastatic disease: CTCs represent the intrapatient tumor heterogeneity of cancer cells residing at multiple metastatic sites.

Recent advances in technology have revolutionized our ability to isolate and characterize CTCs from the blood of patients with numerous forms of cancer (Ferreira *et al*., 2016). The development of these innovative CTC technologies has lead to the concept of using CTCs as a liquid biopsy that may be able to provide prognostic information upon initial diagnosis, track response to therapies, and identify early signs of resistance to treatment (Lianidou *et al*., 2015). Additionally, the analysis of CTCs that are derived from different sites of disease within a patient with metastatic disease has the potential to better detect the intrapatient heterogeneity of the cancer cells compared to a site‐directed biopsy (Fig. 2). The study of CTCs has revealed that markers of EMT are an important feature that can help define and characterize these cells and provide valuable and potentially actionable information to clinicians regarding disease prognosis and response to therapy for individual patients (Satelli *et al*., 2016; Yu *et al*., 2013; Zhao *et al*., 2017). This review will focus on the current state of knowledge regarding EMT in circulating tumor cells.

## 2 Technologies to enrich epithelial and mesenchymal CTCs

### 2.1 Biophysical and functional property‐based enrichment of CTCs

CTCs were first observed in the blood of patients with cancer almost 150 years ago (Ashworth, 1869). They represent a rare population of cells, which are estimated to be present at a frequency of one CTC admixed with a billion blood cells (Maheswaran and Haber, 2010). Therefore, their isolation presents a significant technological challenge. Given their clinical utility as noninvasive blood‐based biomarkers, several platforms have been developed to enrich CTCs from the blood of patients with cancer. Some approaches rely on the functional properties of CTCs including the ability to breakdown collagen and invade or secrete detectable proteins (Deng *et al*., 2014; Lu *et al*., 2010). Other approaches use physical characteristics of the tumor cells including size, density, electric charge, and deformability to distinguish them from blood cells (Hou *et al*., 2013; Liu *et al*., 2015; Mitchell *et al*., 2015; Shaw Bagnall *et al*., 2015). Each method must be evaluated independently to determine the ability to enrich for tumor cells with either epithelial or mesenchymal features (Table 1). For example, there is little difference in the deformability between epithelial and mesenchymal tumor cells, suggesting that CTC enrichment relying on deformability might represent an unbiased approach to capture both CTC lineages (Shaw Bagnall *et al*., 2015). EMT, however, is associated with an increase in cell size (Lamouille and Derynck, 2007), suggesting that size‐based CTC separation methods might be biased toward the enrichment of mesenchymal CTC populations. In fact, size‐based CTC collection from patients with different types of metastatic cancers shows enrichment of CTCs with mesenchymal and stem‐like characteristics (Hou *et al*., 2013; Wu *et al*., 2015). This may reflect increased tumor aggressiveness, but needs to be considered with the caveat that size‐based CTC separation is biased toward enrichment of larger cells.

**Table 1:** Epithelial and mesenchymal circulating tumor cell recovery from methods of CTC isolation

Methods	Detection of Epithelial (E) versus Mesenchymal (M) CTCs	References
Size‐based filtration	E & M	Kim *et al*. (2017), Vona *et al*. (2000)
Density‐based centrifugation	E & M	Liu *et al*. (2015)
Size and deformability	M > E	Gogoi *et al*. (2016)
Cell surface charge	E & M	Gupta *et al*. (2012)
*Negative selection*		
Microfluidic size based followed by negative selection for CD45	E & M	Karabacak *et al*. (2014)
Density separation of tetrameric antibody complexes for CD45, CD66b, and glycophorin	E & M	Naume *et al*. (2004)
*Positive selection*		
Cell surface vimentin	M	Satelli *et al*. (2015a)
High‐throughput microscopy for immunofluorescence or DNA/RNA FISH	E & M	Krivacic *et al*. (2004)
EpCAM‐based immunomagnetic separation	E	Allard *et al*. (2004)
Flow cytometry for surface epithelial markers	E	Gross *et al*. (1995), Magbanua *et al*. (2015)
Filtration using selective size amplification	E	Kim *et al*. (2012)

### 2.2 Antibody‐based CTC isolation

The most common approach underlying CTC isolation technologies is dependent on immunoaffinity purification of tumor cells. In one form of this method, antibodies are directed against proteins that are exclusively expressed on the plasma membrane of the tumor cells and not on hematologic cells resulting in the positive selection of CTCs. Alternatively, antibodies against proteins that are expressed on the surface of the white blood cells can be used to mark and deplete the normal blood components—negative depletion—and the CTCs remaining in the flow‐through can be collected for further analysis.

### 2.3 Positive selection of CTCs

Numerous technologies relying on positive selection of CTCs utilize antibodies against EpCAM, a cell surface glycoprotein expressed on tumor cells of epithelial lineage, but not on leukocytes. CellSearch, the only FDA‐approved CTC isolation device, is based on immunomagnetic separation of CTCs expressing EpCAM and enumeration of the captured cells by staining with a cocktail of antibodies against the epithelial cytokeratins 8, 18, and/or 19 (Andree *et al*., 2016). CellSearch identifies CTCs in about 50% of patients with metastatic disease, which may represent the technical limitation of the technology, or the variable expression of the epithelial markers (Allard *et al*., 2004). Enumeration of CTCs using this technology is prognostic in patients presenting with many types of epithelial cancers, with the presence of ≥5 CTCs per 7.5 mL of blood being predictive of poor clinical outcome (de Bono *et al*., 2008; Hayes *et al*., 2006). CTC technologies including CellSearch, which rely on EpCAM‐mediated capture of CTCs, are limited by their inability to enrich mesenchymal CTCs which either express low levels or no EpCAM. CTCs that fail to be captured by EpCAM‐dependent methods are detectable with antibodies directed against mesenchymal as well as other tumor markers (Raimondi *et al*., 2011; Schneck *et al*., 2015), suggesting that CTCs are indeed heterogeneous for epithelial and mesenchymal markers. To overcome the limitation of EpCAM‐mediated CTC enrichment and to capture CTCs irrespective of their EMT status, antibody cocktails against EGFR and HER2 (tumor‐specific markers), CDH11 (a cadherin specifically overexpressed in mesenchymal tumor cells), as well as cell surface vimentin (expressed in cancer cells of mesenchymal lineage) (Armstrong *et al*., 2011; Satelli *et al*., 2015a; Yu *et al*., 2013), have been used in combination with EpCAM antibodies. Importantly, broadening the spectrum of detection antibodies beyond those solely against epithelial cytokeratins has improved the identification of mesenchymal CTCs in the blood (Armstrong *et al*., 2011; Satelli *et al*., 2015a; Yu *et al*., 2013).

### 2.4 Negative depletion of leukocytes

The efficiency of positive selection technologies is significantly impeded by the extensive heterogeneity exhibited by tumor cells in the blood. The negative depletion approach based on the removal of normal blood components circumvents this problem leading to antigen‐independent purification of CTCs. This minimizes the loss of CTC populations, whose heterogeneity can be attributed to various determinants including EMT. As highly specific antibodies with high affinity against mouse leukocyte populations are readily available, the negative selection approach can also be readily applied to isolate CTCs from mouse tumor models, negating the search for mouse‐specific antibodies required for positive selection of these CTCs (Ting *et al*., 2014). However, this approach may also enrich nonhematopoietic cells such as circulating endothelial cells as well as other circulating epithelial cells (Goon *et al*., 2006). Thus, CTC identification and enumeration following negative depletion requires carefully chosen epithelial and mesenchymal markers benchmarked against healthy donor blood, as well as molecular analyses that ensure their tumoral origin (Yu *et al*., 2013). Nevertheless, staining CTCs with epithelial and mesenchymal markers following negative depletion shows that CTCs in the blood are comprised of both epithelial and mesenchymal tumor cells (Yu *et al*., 2013).

## 3 Functional characteristics of epithelial and mesenchymal CTC populations

### 3.1 EMT of epithelial cancers

In addition to the significant advances in the ability to isolate CTCs, a second important advance in the study of CTCs is the development of analytical tools including single‐cell RNA‐Seq (Saliba *et al*., 2014; Tang *et al*., 2010), multicolor RNA *in situ* hybridization (Wu *et al*., 2016), and single‐cell DNA methylation analysis available to characterize CTCs (Pixberg *et al*., 2017). While still in the development stages, the potential rapid advancement of single‐cell proteomics will likely also prove to be a critical tool (Macaulay *et al*., 2017). The single or clustered nature of CTCs and their rarity necessitates the use of these technologies; however, the analysis of CTCs with these technologies has provided important insight into the biology of CTCs including their heterogeneity. Future technologic advancements will permit the multimodality analysis of the same single cell to give a richer view of CTC biology.

RNA *in situ* hybridization (RNA‐ISH) using multiple probes to mark epithelial and mesenchymal states has been applied to CTCs to increase detection sensitivity without compromising specificity (Yu *et al*., 2013). A dual colorimetric RNA‐ISH using two pools of probes, one against seven epithelial markers (keratins (KRT) 5, 7, 8, 18, and 19; EpCAM, and E‐cadherin) and the other against three mesenchymal markers [(FN1 (fibronectin 1), CDH2 (cadherin 2), and SERPINE1/PAI1 (serpin peptidase inhibitor, clade E)], was used to visualize breast CTCs isolated by positive selection using antibodies against HER2, EGFR, and EpCAM. Semiquantitative measurement of the punctate epithelial and mesenchymal RNA‐ISH signals can define the degree of epithelial and mesenchymal characteristics of each individual CTC. These results showed that CTCs exhibit varying degrees of epithelial and mesenchymal characteristics (e.g., epithelial (E), mesenchymal (M), E > M, E = M, and M > E), demonstrating that EMT is a continuous process. In fact, the biphenotypic tumor cells simultaneously expressing both epithelial and mesenchymal markers may represent the most plastic and potentially the cells most likely to contribute to metastatic outgrowth (Jolly *et al*., 2015). The EMT status of CTCs in patients with breast cancer is dependent on the breast cancer subtype: Mesenchymal CTCs are prevalent in triple‐negative and HER2‐positive breast cancer patients, whereas CTCs in lobular breast cancer patients are predominantly epithelial (Yu *et al*., 2013). Lobular breast carcinoma is marked by mutations or loss of E‐cadherin, a critical epithelial protein. Yet, tumor cells within the primary tumor or metastatic lesion do not exhibit complete conversion to a mesenchymal state although these tumors are marked by an invasive growth pattern (McCart Reed *et al*., 2016). Analysis of tumor cells invading through the blood is consistent with these findings. Further studies are needed to continue to define what elements of epithelial plasticity contribute to metastatic spread and potentially the cooperative interactions between cells with different phenotypes.

In addition to breast cancer, heterogeneity of EMT markers in CTCs has also been observed in other cancers. RNA‐ISH analysis of CTCs isolated from patients with liver, nasopharyngeal, breast, colon, gastric, and non‐small‐cell lung cancer showed the presence of epithelial, biphenotypic, and mesenchymal populations (Wu *et al*., 2015). The ratio of mesenchymal CTCs increased based on the TNM stage of the cancer for all of the cancers analyzed (Wu *et al*., 2015). A separate study of a mouse model of pancreatic cancer also identified varying degree of EMT in CTCs (Rhim *et al*., 2012). Interestingly, cells exhibiting mesenchymal markers are also seen in premalignant pancreatic intraepithelial neoplasms and in circulating pancreatic‐derived cells prior to the development of overt cancer. Therefore, EMT and malignant cell dissemination may occur early in the development of cancer. Additionally, while tumor cells displaying mesenchymal markers are not significantly enriched in the CTCs, functional analysis revealed that these cells had increased tumor‐initiating potential. Together, these results support the concept of epithelial and mesenchymal heterogeneity in CTCs and provide evidence that EMT may not only permit invasion and escape from the primary tumor, but may induce increased tumor‐initiating properties.

Analysis of epithelial and mesenchymal CTC populations in a cohort of patients with breast cancer showed a dynamic shift in the populations during treatment, such that increasing number of mesenchymal CTCs correlated with treatment failure and disease progression (Yu *et al*., 2013). Increase in the mesenchymal CTC population during relapse is associated with the appearance of tumor emboli or multicellular CTC clusters in the blood. The CTC clusters are highly mesenchymal and are not readily detectable with antibodies against epithelial cytokeratins. The mesenchymal state of the CTC clusters may be attributed to the coating of platelets, which are a rich source of TGF‐β, a potent inducer of EMT (Labelle *et al*., 2011). Indeed, transcriptome analysis of the breast CTC clusters shows the enrichment of TGF‐β and EMT signatures, suggesting that CTC clusters, which are oligoclonal and held together through cell–cell interactions, most likely arise through collective migration of tumor cells, a process dependent on partial EMT (Aceto *et al*., 2014; Friedl and Gilmour, 2009; Yu *et al*., 2013).

RNA sequencing of single CTCs has also enabled the comprehensive analysis of these cells and has been used to interrogate the EMT characteristics of CTCs in mouse tumor models as well as in patients. The microfluidic device, the iChip, which removes red blood cells and platelets through size‐based separation, enriches CTCs by magnetophoretic depletion of immunomagnetically tagged white blood cells (Karabacak *et al*., 2014). These CTCs collected in solution can be picked as single cells using a micromanipulator and analyzed with RNA‐Seq at single‐cell resolution (Miyamoto *et al*., 2015; Ting *et al*., 2014). This approach was applied to CTCs isolated from a genetically engineered mouse model of pancreatic cancer [(LSL‐Kras^G12D^, Trp53^flox/flox or +^, PDX‐Cre (KPC)], which closely mimics the progression of human pancreatic cancer. Single‐cell RNA‐Seq performed on individual CTCs identified three distinct populations: CTCs expressing epithelial markers (classical CTCs—CTC‐c), CTCs enriched for platelet markers (CTC‐plt) and CTCs with a robust proliferation signature (CTC‐Pro) (Ting *et al*., 2014). The classical CTCs, in comparison with tumor cells within the primary tumor, exhibited a universal loss of E‐cadherin, demonstrating their propensity to lose epithelial markers. This is consistent with the concept of EMT contributing to the invasion of these mouse pancreatic tumor cells into the stroma and blood (Rhim *et al*., 2012). However, the expression of mesenchymal genes in these CTCs is more complex and highly variable. When compared to the primary tumor, the expression of some EMT markers, CDH11 and vimentin, is elevated in CTCs, whereas others, S100A4, Itga5, Sdc1, are decreased. The heterogeneity exhibited by CTCs in these mice is consistent with the continuity and complexity associated with the EMT process and the presence of several intermediate EMT states in CTCs in human cancer patients. The transition of epithelial cancer cells to a mesenchymal state has been shown to be closely associated with the acquisition of stem‐like characteristics (Guo *et al*., 2012; Mani *et al*., 2008). Interestingly, the mouse pancreatic CTCs expressed high levels of Aldh1a1 and Aldh1a2 mRNA, but the expression of these stemness markers did not correlate with EMT, suggesting that these phenotypes are not linked in pancreatic CTCs. The absence of a correlation between the mesenchymal and stemness markers is also observed in CTCs isolated from castration‐resistant prostate cancer patients (Miyamoto *et al*., 2015).

A transgenic mouse model of prostate cancer tumorigenesis and metastasis was paired with a vimentin‐GFP reporter to track the expression of this mesenchymal marker in prostate tumor cells (Ruscetti *et al*., 2015). Analysis of the peripheral blood of these mice at different stages of tumorigenesis revealed an increase in mesenchymal (EpCAM−/GFP+) and biphenotypic (EpCAM+/GFP+) CTCs correlating with metastatic disease. Interestingly, while the mesenchymal and biphenotypic CTCs have increased tumor‐initiating ability in the prostate, only the biphenotypic and epithelial CTCs could form macrometastases. The mesenchymal CTCs persisted as micrometastatic foci. These results support the idea that epithelial plasticity is critical to metastasis. However, it is important to point out that in a study of prostate cancer CTCs derived from patients with metastatic disease, RNA‐Seq data of single cells did not identify downregulation of epithelial markers or upregulation of mesenchymal markers in subpopulations of the CTCs (Miyamoto *et al*., 2015), suggesting either that the heterogeneity can be lost in a moderately pretreated cohort or that epithelial and mesenchymal heterogeneity is not well represented in single‐cell RNA‐Seq analysis. Further studies are needed to investigate the presence of epithelial and mesenchymal marker heterogeneity in patient‐derived prostate samples.

### 3.2 EMT signatures in CTCs from nonepithelial tumors

Besides epithelial tumors, enrichment of EMT signatures is also observed in CTCs derived from nonepithelial tumor types, including melanoma and glioblastoma. Loss of E‐cadherin is observed in late‐stage malignant melanoma (Alexaki *et al*., 2010; Miller and Mihm, 2006) and the EMT‐inducing transcription factor network significantly changes during melanomagenesis and constitutes a risk factor for poor outcome in malignant melanoma (Caramel *et al*., 2013). The expression of EMT‐associated genes is a major determinant of melanoma metastasis (Alonso *et al*., 2007), suggesting that this pathway might be involved in promoting melanoma dissemination. Indeed, RNA‐Seq analysis of CTCs isolated from a B‐RAF/PTEN‐mutant mouse melanoma model, compared with primary tumor cells, shows the upregulation of EMT signatures coincident with genes implicated in tumor invasiveness (Luo *et al*., 2014). Similarly, glioblastoma CTCs isolated from patients as well as from a PDX mouse model, when compared with their matched parental tumor, show the upregulation of RNA encoding for the mesenchymal genes SERPINE1, TGFB1, TGFBR2, and vimentin. Overexpression of EMT regulators is observed in the mesenchymal subset of GBMs and is associated with therapeutic resistance and poor clinical outcome (Bhat *et al*., 2013; Colman *et al*., 2010; Phillips *et al*., 2006).

### 3.3 Potential genomic changes induced by EMT

The plasticity of EMT in epithelial cancer cells is generally attributed to epigenetic changes which are reversible (Bedi *et al*., 2014; McDonald *et al*., 2011). During development, proliferation is strictly restricted in gastrulating cells undergoing EMT; initiation of proliferation in these cells during EMT leads to severe developmental defects (Murakami *et al*., 2004; Seher and Leptin, 2000). The incompatibility between EMT and proliferation is also observed in cancer cells. TGF‐β and SNAIL‐induced EMT in proliferating mammary epithelial cells are associated with mitotic defects leading to binucleate cells and extensive chromosome missegregation (Comaills *et al*., 2016). These mitotic defects are mediated through TGF‐β and SNAIL‐mediated suppression of multiple nuclear envelope proteins including nuclear lamins, which maintain the nuclear architecture and orchestrate nuclear transport as well as mitotic processes. The mitotic aberrations induced during EMT are reversible; however, the resulting genomic instability leads to heritable changes. Single‐cell RNA‐Seq analysis of breast and prostate CTCs shows a correlation between TGF‐β and EMT signatures and aneuploidy. Furthermore, binucleated and micronuclei harboring cells were prevalent in CTCs of mesenchymal lineage compared to epithelial lineage, suggesting that EMT might be a mechanism leading to genomic instability. These findings that are consistent with developmental models (Murakami *et al*., 2004; Seher and Leptin, 2000) might explain the drug resistance associated with EMT (Maheswaran and Haber, 2015; Nieto *et al*., 2016). In fact, TGF‐β activation in the CD44+/CD24− stem‐like cells increases DNA copy number alterations and contributes to changes in drug responses (Pal *et al*., 2017). Taken together, these findings suggest that EMT in proliferating cancer cells could be a major contributor to the molecular evolution and heterogeneity of tumors during disease progression.

## 4 Clinical implications of epithelial‐to‐mesenchymal transition in circulating tumor cells

EMT provides a useful model to understand the mechanisms underlying tumor cell invasion and motility, and CTCs offer the possibility of capturing tumor cells during an intermediate step of cancer metastasis; therefore, the overlap of these two concepts will provide a more comprehensive understanding of metastasis. Importantly, though, the study of EMT in CTCs likely has clinical implications that are key to the development of novel cancer treatments and improved deployment of our current therapies. Markers of EMT in CTCs have the potential to provide both prognostic and predictive information. As discussed above, in breast cancer, the number of mesenchymal CTCs has been linked to disease progression and decreased overall survival (Polioudaki *et al*., 2015; Yu *et al*., 2013). In colorectal cancer, a study of CTCs in over a thousand patients found that biphenotypic and mesenchymal CTCs correlated with clinical stage, lymph node, and distant metastases (Zhao *et al*., 2017). Interestingly, a study of metastatic colorectal and prostate cancer patients observed that cell surface vimentin‐positive CTCs that expressed nuclear programmed death‐ligand 1 (PD‐L1) are associated with decreased survival, suggesting that there may exist subpopulations within the mesenchymal CTCs that correlate with clinical parameters (Satelli *et al*., 2016). In addition to potential prognostic markers of disease, markers of EMT in CTCs also have the potential to provide predictive information regarding a patient's response to therapy. Currently, this area of research is in its early stages. For example, in a recent clinical study of metastatic colorectal cancer, enumeration of CTCs with cell surface vimentin correlated with disease response to postsurgery chemotherapy, suggesting that markers of EMT in CTCs may also prove to be useful markers to predict response to therapy (Satelli *et al*., 2015b). In a preclinical model of breast cancer metastasis, FOXC2 has been identified as an inducer of EMT and an important regulator of metastasis (Werden *et al*., 2016). FOXC2 induction of EMT is dependent on MAP kinase activity, and in this study, an inhibitor of the p38 MAP kinase inhibited metastatic spread induced by FOXC2. Therefore, FOXC2 in CTCs may represent a potential marker predicting response to MAP kinase inhibitors. Future studies and investigation of additional markers will be needed.

## 5 Future directions

The study of EMT and the parallels between development and cancer have provided significant insight into the metastatic process. The recent advancements in the isolation and characterization of CTCs provide novel tools and information about how EMT contributes to cancer spread. However, one of the challenges of the current studies of both EMT and CTCs is the lack of standardization. For instance, the classification of epithelial, mesenchymal, and biphenotypic cells varies depending on the study even within the same cancer type. Similarly, the isolation of CTCs varies significantly based on the method and the markers used to purify these cells. It will be important to standardize the classifications and methods in order to increase the robustness of our current knowledge. With this in mind, it will be important to reliably establish the degree of heterogeneity of CTCs and clearly define their subpopulations. A consistent isolation and classification method will permit the accurate tracking of changes in CTCs and allow for testing of potential biomarkers of disease progression or response to therapy. Once biomarkers are identified and a reliable method of detection is established, prospective trials testing these biomarkers will be required to establish their clinical usefulness and validity. In addition to the focus on clinical tools based on EMT in CTCs, improved single‐cell technologies will continue to advance our understanding of CTCs. For example, it is likely that the expression of mesenchymal genes and misexpression of developmental EMT regulators are accompanied by epigenetic changes that activate these typically repressed genes. Epigenetic regulators are potential novel drug targets that offer a unique mechanism of action that could alter the cellular plasticity. Future investigations will need to further define the role of EMT in CTC generation, survival, extravasation, and colonization of distant organs with the hope of developing new therapies and using our current therapy more effectively.
